# Phenytoin-Induced Toxic Epidermal Necrolysis

**DOI:** 10.7759/cureus.42654

**Published:** 2023-07-29

**Authors:** Mounika Nagarani Tunuguntla, Pranathi Chanti, Tanishq Kesani, Kusuma Yarapathineni, Prithvi Kukkadapu

**Affiliations:** 1 Internal Medicine, Guntur Medical College, Guntur, IND; 2 Internal Medicine, Osmania Medical College, Hyderabad, IND; 3 Internal Medicine, Siddhartha Medical College, Vijayawada, IND; 4 Internal Medicine, Huntsville Hospital, Huntsville, USA

**Keywords:** skin lesions, exfoliative dermatitis, keratinocyte necrosis, severe cutaneous adverse reaction, steven-johnson syndrome (sjs), sjs/ten, phenytoin, cyclosporine for ten, ten

## Abstract

Toxic epidermal necrolysis (TEN) is a rare fatal mucocutaneous blistering disorder that can have varied underlying triggers. The percentage of body surface area (BSA) that is impacted by erosive blistering is what separates it from Steven Johnson syndrome (SJS), both of which have the same underlying pathogenesis and are thought to exist on a continuum of disease with TEN being the more serious of the two. Medications are the most frequent cause of TEN/SJS and typically cause disease in both adults and children within eight weeks; however, the median exposure window is four days to four weeks. Nonsteroidal anti-inflammatory drugs, allopurinol, anticonvulsants including lamotrigine, phenytoin, levetiracetam and carbamazepine, antimicrobial sulfonamides, and the antiviral nevirapine are examples of medications that frequently cause TEN/SJS. Here, we are reporting a case of phenytoin-induced TEN highlighting the patient's excellent response to immunomodulating treatment despite 100% involvement of the BSA.

## Introduction

Toxic epidermal necrolysis (TEN), a severe cutaneous adverse reaction (SCAR) that is characterized by keratinocyte necrosis with separation of the epidermis from the underlying dermis, occurs typically as a result of an abnormal immune response to specific medications and less commonly following certain infections. Here, we are presenting a case of TEN induced by phenytoin. This case report aims to highlight the excellent response to systemic immunomodulating therapy with cyclosporine and dexamethasone despite the involvement of 100% or total body surface area (TBSA) and provide a review of the multifaceted approach to the management of TEN. 

## Case presentation

A 40-year-old female, with a known history of epilepsy since childhood, who has been incompliant with her maintenance therapy of levetiracetam, presented to the emergency department following five episodes of generalized tonic-clonic seizures (GTCS). These lasted for five minutes each, with the involuntary passage of stool and urine. The patient was started on injection phenytoin and injection levetiracetam.

**Figure 1:**
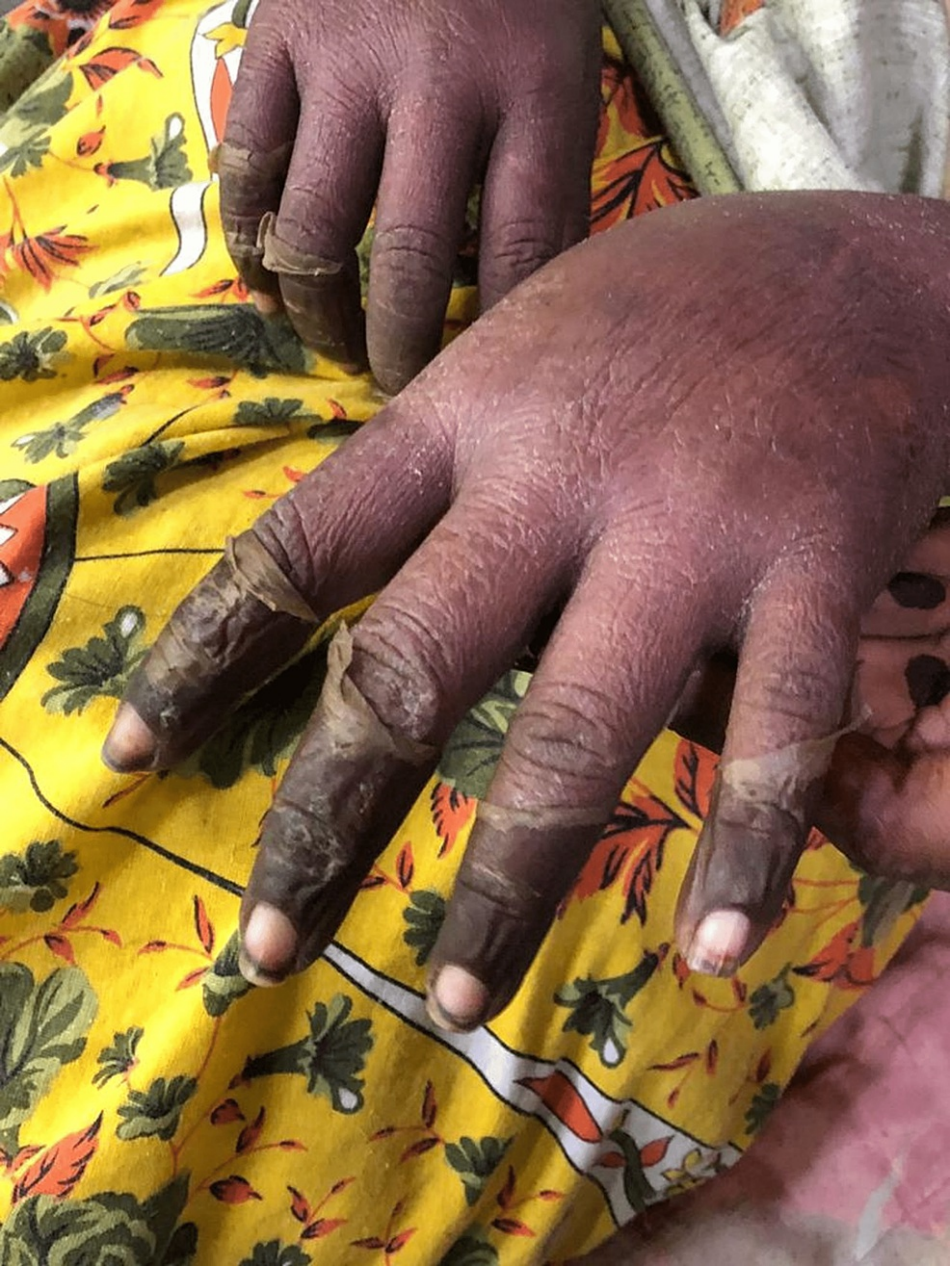
Peeling of skin over the hands

Five days after starting phenytoin, the patient started to develop a high-grade fever of 103°F, diffuse painful skin peeling throughout the body (Figure 1), also involving mucosal areas like the mouth, which led to painful swallowing. Skin lesions continued to progress throughout the body, involving TBSA.

**Figure 2:**
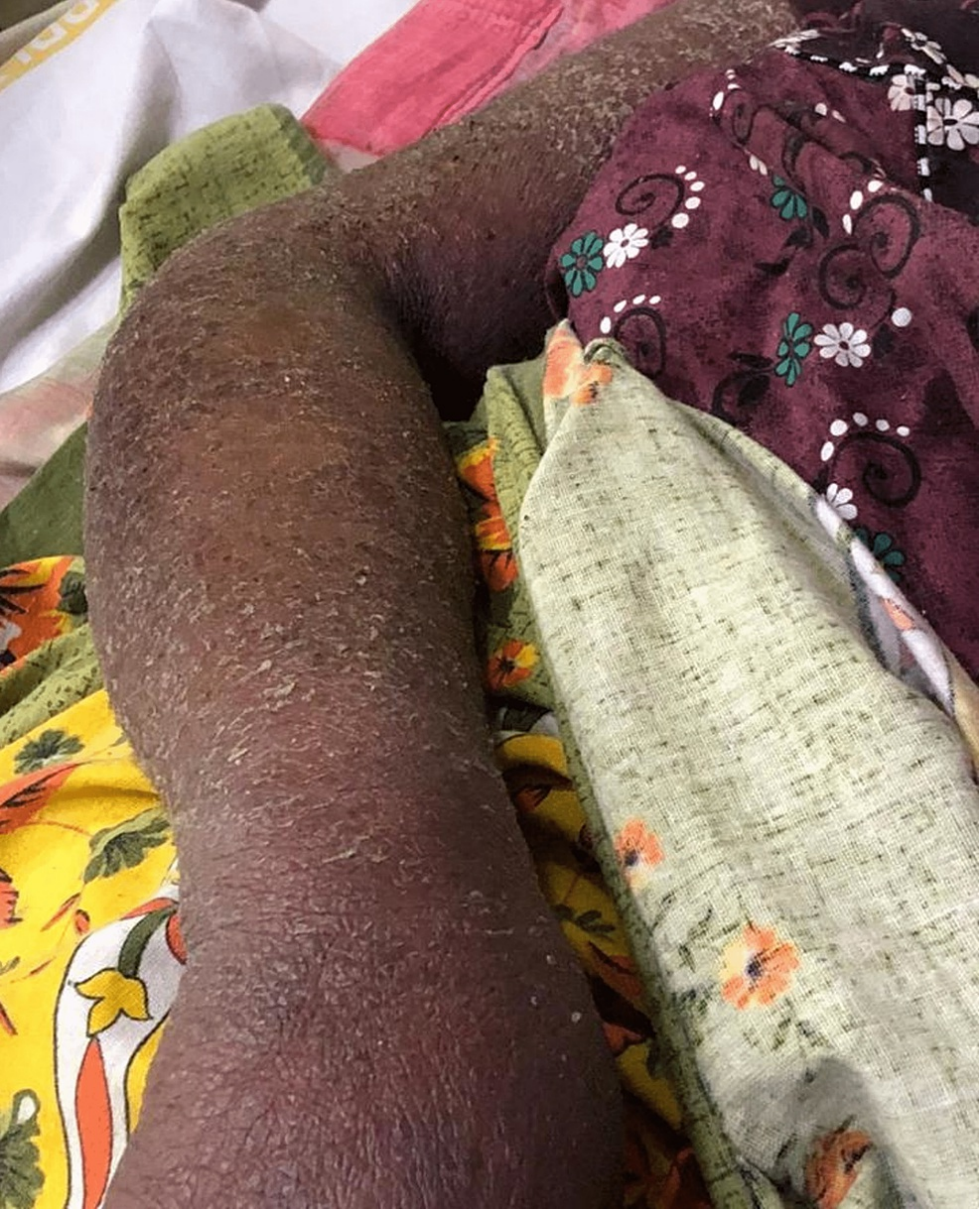
Exfoliative dermatitis involving the arm

**Figure 3:**
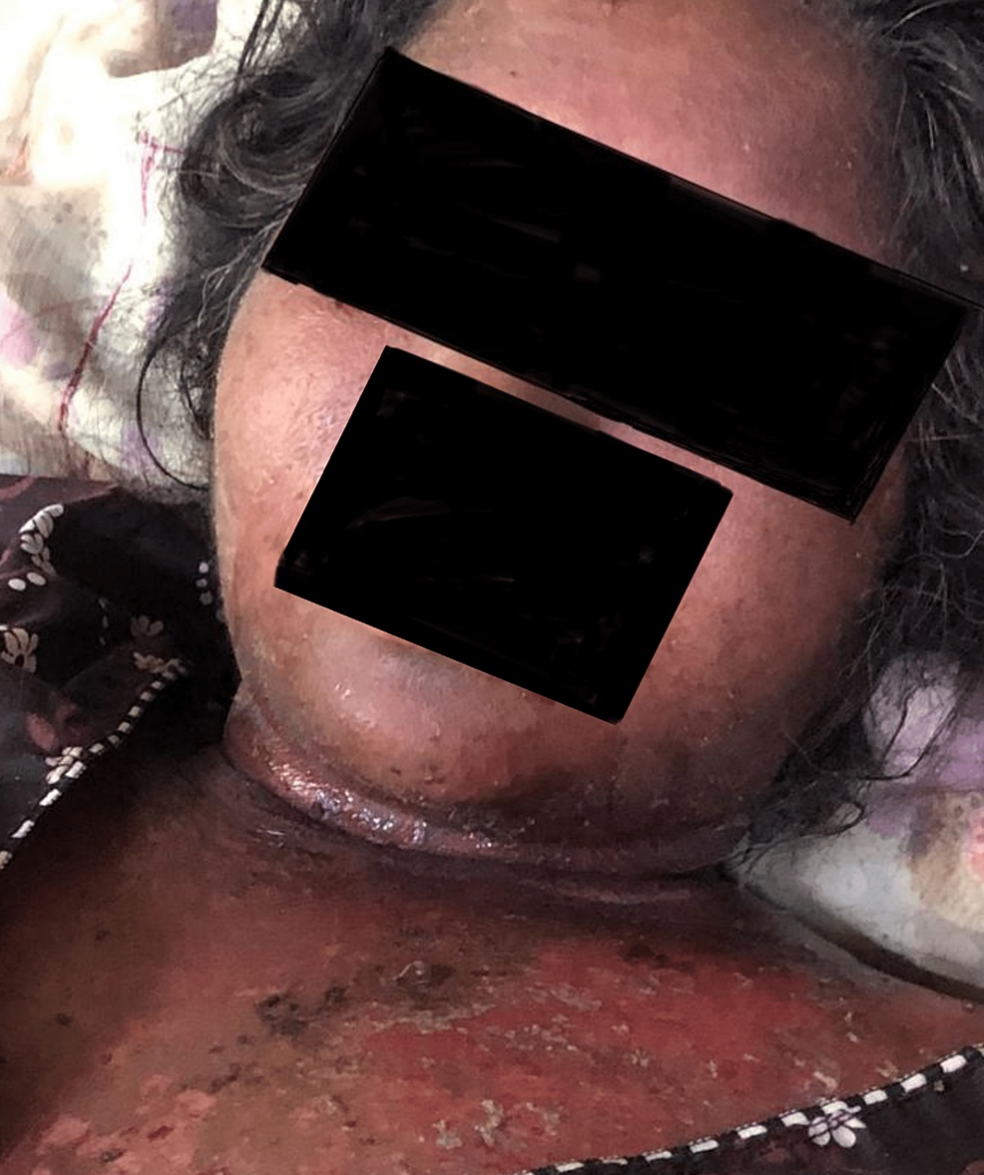
Exfoliative dermatitis over neck and chest

The patient had a history of similar skin lesions 10-12 years back following hospitalization for an episode of seizure due to some unknown medication, likely an antiepileptic drug; the skin lesions occurred after she was given treatment for her epilepsy. On examination, diffuse skin peeling and erythroderma (Figures 2, 3) were noted throughout the body, with oral mucosa showing erosions.

A clinical diagnosis of TEN was made, and an immediate review of all the medications that the patient had been kept on was done, after which phenytoin was discontinued. Casualty assessment by the Naranjo scale suggested probable adverse drug reactions (ADR), which means that the reaction followed a reasonable temporal sequence after a drug, followed a recognized response to the suspected drug, was confirmed by withdrawal but not by exposure to the drug, and could not be reasonably explained by the known characteristics of the patient’s clinical state. Skin lesions continued to progress throughout the body, involving TBSA.

Baseline investigations were significant for leucopenia where the total white blood cell count was 1900 cells/cumm and neutropenia where the absolute neutrophil count was 570 cells/cumm. Serum glucose was 290 mg/dl. Liver function tests revealed elevated serum glutamic oxaloacetic transaminase (SGOT) and serum glutamic pyruvic transaminase (SGPT) levels of 101 IU/L and 51 IU/L, respectively, with reference ranges being up to 40 IU/L for both SGOT and SGPT and low total proteins of 5.2g/dl (Reference value 6.2-8.8 g/dl) and albumin of 3 g/dl (Reference value 3.5-5.1 g/dl). Renal function tests were significant for a slightly elevated blood urea nitrogen of 48 mg/dl (Reference value 10-45 mg/dl) and serum creatinine of 1.2 mg/dl (Reference value 0.6-1.5 mg/dl). Serum electrolytes were found to be in their normal range.

The patient was started on dexamethasone and cyclosporine to stop further damage. Fluid and electrolyte balance was maintained by aggressive intravenous fluids along with proper antibiotic coverage. Skin lesions were managed with the local application of Soframycin cream and liquid paraffin over the raw areas to prevent infection and excessive loss of water and electrolytes through the defective skin barrier. The patient was also put on a high-protein diet with a calorie intake of around 3000 calories/day. The hospital course, however, was complicated by pneumonia, for which the patient required non-invasive ventilation (NIV).

Over the next week, the patient's skin lesions started to heal and recovered from her pneumonia. She was successfully discharged after 12 days.

## Discussion

TEN was first described by Lyell. Hence it is also known as Lyell's syndrome. The estimated overall incidence of TEN varies from two to seven cases per million with a 1000-fold increased incidence seen in HIV-positive patients [1].

TEN is one of the SCARs, the others being Steven-Johnson syndrome (SJS), drug-induced hypersensitivity syndrome (DIHS), drug reaction with eosinophilia and systemic symptoms (DRESS), and acute generalized exanthematous pustulosis (AGEP). The only significant difference between SJS and TEN is the extent of involvement. SJS involves less than 10% of BSA, while TEN involves more than 30% of BSA. The involvement of 10-30% of BSA is categorized as SJS/TEN overlap.

SJS/TEN typically manifests within eight weeks of the introduction of the inciting agent, most commonly however occurring four days to four weeks after the inciting agent has been introduced [2].

A lot of drugs and infections have been implicated in the causation of SJS/TEN in a genetically susceptible patient Almost any drug can cause TEN/SJS, with antibiotics, anticonvulsants, and anti-inflammatory medications being the most frequently encountered. Infections caused by *Mycoplasma pneumoniae*, herpes, and hepatitis A can also lead to TEN/SJS.

Severity of illness score in TEN (SCORTEN) uses age, associated cancer, heart rate, serum blood urea nitrogen, serum bicarbonate, serum glucose, and detached BSA to calculate the risk of mortality [2]. In our case, the SCORTEN was been found to be 4.

A lot of theories have been proposed for explaining the underlying pathophysiology of SJS/TEN like the hapten/prohapten concept, and the p-i concept (pharmacology with immunology). Haptens are small chemicals that are too small to be antigenic by themselves and to elicit an immune response. However, due to their innate nature of chemical reactivity, they bind to other proteins in the body which could theoretically be any protein including albumin, transmembrane proteins, or even intracellular proteins. They tend to bind to these and induce a conformational change of those proteins leading to the formation of a neoantigen which becomes antigenic. The hapten protein complex so formed binds to the major histocompatibility complex (MHC) class two on the antigen-presenting cells and gets presented to the T cell leading to the activation of T cells setting up an immune response [3].

Not all drugs are chemically reactive by themselves. The ability of those drugs to cause SJS/TEN or in fact any other reaction is explained by other theories such as the prohapten concept and the p-i concept. Prohapten concept simply states that though the drug by itself is chemically unreactive, it becomes antigenic after it's converted to other metabolites in the body that could then act as a hapten eliciting an immune response

p-i concept, which is the pharmacological interaction of drugs with immune receptors, is another theory that tries to explain how chemically unreactive drugs are able to elicit an immune response. It states that drugs could directly bind to MHC molecules or even the T cell receptors directly to elicit an immune response [4].

A phase of prodromal symptoms typically precedes the skin lesions, often including fever, malaise, cough, and sore throat in most cases. Skin lesions initially appear as erythematous macules or atypical target lesions which then progress to form erosions and flaccid blisters, and subsequently result in the peeling off of the skin, leaving portions of the body denuded. The cleft in TEN blisters is only subepithelial, resulting in a positive Nikolsky sign, where the affected skin detaches even with gentle lateral pressure.

TEN often involves mucosal surfaces, with more than 80% of cases showing involvement of two or more mucosal surfaces [2], like the oral mucosa, ocular mucosa, gastrointestinal mucosa, and genital mucosa.

The course of SJS/TEN can be affected by a lot of complications. Acute complications are secondary bacterial infection of the lesions with potential for sepsis, dehydration, electrolyte imbalances, protein loss and malnutrition, conjunctivitis, and keratitis in case of ocular involvement, rarely, death all of which are occurring due to the loss of natural barrier function of the skin and the mucosa which prevents infections and excessive water and electrolyte loss. Involvement of tracheal or bronchial epithelium is seen in up to 20% of the cases and leads to severe complications [5]. However, the mortality rate has been decreasing due to recent improvements in the management of TEN/SJS. Long-term sequelae are common and include hyper and hypopigmentation of the skin and nail dystrophies [6]. Yip et al. concluded that ocular complications occur in half of the patients and can lead to dry eyes, trichiasis, symblepharon, distichiasis, visual loss, entropion, ankyloblepharon, lagophthalmos, and corneal ulceration in the descending order of occurrence [7]. Other chronic sequela to SJS/TEN due to mucosal involvement include strictures involving the esophagus, intestines urethra, and anus.

Management of a patient with TEN is multifaceted and is aimed towards stopping further skin damage, local care of the skin and mucosa, and accelerating their regeneration, preventing complications, or treating them if they have already occurred. A detailed history of all the drugs that the patient has been taking is crucial since the manifestations of TEN occur within eight weeks of starting the drug with most cases occurring between four days and four weeks of starting the drug. ALDEN (algorithm for drug causality for epidermal necrolysis) is an algorithm that can be used if the culprit is not readily identifiable by history. The use of any drugs, especially those taken within one to eight weeks of the TEN manifestation, that are likely to have contributed to the condition must be discontinued [8].

A recent systematic review and meta-analysis by Zimmermann et al. concluded that glucocorticoids and cyclosporin are the most promising choice as a part of systemic immunomodulating treatment [9], which has shown excellent results in our case where skin lesions started to improve drastically over a period of a week with the administration of cyclosporine and steroids. Cyclosporine, which is a selective T-cell inhibitor, reduces the time to stop the disease from progressing and also the time required for epithelial regeneration [10].

Local care of skin and mucosa is essential to fasten the regeneration of the skin and mucosa and to prevent both short-term complications and also long-term sequelae, especially those that are involving the mucosa. Liquid paraffin/white soft paraffin (50:50) can be applied to the denuded areas to prevent further loss of water through the defective skin barrier. Antiseptics like chlorhexidine 0.05% or silver nitrate 0.5% could be used to paint or for dressings. It is important to use sulfa-containing drugs cautiously, especially when those are the prime suspects. Dressings are considered beneficial in these patients and have shown better outcomes in terms of sepsis control and faster re-epithelisation rates [6]. Non-adhesive dressings with silver nitrate 0.5% or polyvidone iodine can be used to cover denuded areas. Nanocrystalline silver dressing (NCS) is an excellent type of dressing that should be used due to its potent antibacterial effect, anti-inflammatory effect, and lesser need for frequent change of the dressing [11].

## Conclusions

TEN is a life-threatening condition requiring prompt recognition, immediate cessation of the drug, and appropriate management with immunosuppressants if severe enough. Educating the patient about their hypersensitivity to a particular drug could potentially prevent recurrent episodes to the same drug in the future.
